# Comparative Metabolite Profile, Biological Activity and Overall Quality of Three Lettuce (*Lactuca sativa* L., Asteraceae) Cultivars in Response to Sulfur Nutrition

**DOI:** 10.3390/pharmaceutics13050713

**Published:** 2021-05-13

**Authors:** Muna Ali Abdalla, Fengjie Li, Arlette Wenzel-Storjohann, Saad Sulieman, Deniz Tasdemir, Karl H. Mühling

**Affiliations:** 1 Institute of Plant Nutrition and Soil Science, Kiel University, Hermann-Rodewald-Str. 2, 24118 Kiel, Germany; ssulieman@plantnutrition.uni-kiel.de; 2 GEOMAR Centre for Marine Biotechnology (GEOMAR-Biotech), Research Unit Marine Natural Products Chemistry, GEOMAR Helmholtz Centre for Ocean Research Kiel, Am Kiel-Kanal 44, 24106 Kiel, Germany; fli@geomar.de (F.L.); awenzel-storjohann@geomar.de (A.W.-S.); dtasdemir@geomar.de (D.T.); 3 Faculty of Mathematics and Natural Sciences, Kiel University, Christian-Albrechts-Platz 4, 24118 Kiel, Germany

**Keywords:** lettuce, hydroponics, sulfur, sesquiterpene lactone, antioxidant, antibacterial activity, cyanidin 3-*O*-galactoside, flavonoids, organic acids, sugars

## Abstract

The main objective of the present study was to assess the effects of sulfur (S) nutrition on plant growth, overall quality, secondary metabolites, and antibacterial and radical scavenging activities of hydroponically grown lettuce cultivars. Three lettuce cultivars, namely, Pazmanea RZ (green butterhead, V1), Hawking RZ (green multi-leaf lettuce, V2), and Barlach RZ (red multi-leaf, V3) were subjected to two S-treatments in the form of magnesium sulfate (+S) or magnesium chloride (−S). Significant differences were observed under −S treatments, especially among V1 and V2 lettuce cultivars. These responses were reflected in the yield, levels of macro- and micro-nutrients, water-soluble sugars, and free inorganic anions. In comparison with the green cultivars (V1 and V2), the red-V3 cultivar revealed a greater acclimation to S starvation, as evidenced by relative higher plant growth. In contrast, the green cultivars showed higher capabilities in production and superior quality attributes under +S condition. As for secondary metabolites, sixteen compounds (e.g., sesquiterpene lactones, caffeoyl derivatives, caffeic acid hexose, 5-caffeoylquinic acid (5-OCQA), quercetin and luteolin glucoside derivatives) were annotated in all three cultivars with the aid of HPLC-DAD-MS-based untargeted metabolomics. Sesquiterpene lactone lactucin and anthocyanin cyanidin 3-*O*-galactoside were only detected in V1 and V3 cultivars, respectively. Based on the analyses, the V3 cultivar was the most potent radical scavenger, while V1 and V2 cultivars exhibited antibacterial activity against *Staphylococcus aureus* in response to S provision. Our study emphasizes the critical role of S nutrition in plant growth, acclimation, and nutritional quality. The judicious-S application can be adopted as a promising antimicrobial prototype for medical applications.

## 1. Introduction

Sulfur (S) is a macronutrient required for plant growth and metabolism. In plants, it is very important for the synthesis of proteins, enzymes, vitamins, and chlorophyll. Consequently, S influences the growth, development, nutritional quality, and disease tolerance or resistance of the plant [1,2,3,4]. S is the third most abundant mineral element found in the human body after calcium and phosphorus. It represents ~0.3% of total body mass and plays an essential role in the synthesis of important metabolic intermediates, including glutathione [5]. Most dietary S is supplied by proteins, and only two of the 20 amino acids (methionine and cysteine) that are usually present in proteins contain S. The defined requirement for an adult male is 14 mg/day per kg body weight [6]. Notably, S bonds such as disulfide bonds have received considerable attention in biochemical, pharmaceuticals, and biotechnological fields. They have fascinating characteristics, including the potential to break into a reduced glutathione via a thiol–disulfide exchange reaction, in addition to their stability in the human body, with no physiological toxicity [7].

Lettuce (*Lactuca sativa* L., Asteraceae) is widely considered as the most important leafy vegetables in the world and is known as the most common fresh-cut vegetable [8]. North America and Europe were originally the dominant markets for lettuce. However, by the late 20th century, the consumption of lettuce had spread throughout the world. Interestingly, the world production of lettuce and chicory was 26.1 million tons in 2015, of which approximately 56% was produced in China [9]. The global hydroponic vegetables market is expected to increase from US$6934.6 million in 2016 to US$12,106.5 million by the end of 2025 [10]. Importantly, of the various vegetables, the lettuce segment is projected to lead the global market because it is poised to expand at a CAGR (compound annual growth rate) of 6.6% during the forecast period [10]. There is a common conception that fresh vegetables are always superior in nutritional value. Lettuce is known for it is low calories, fat, and sodium content. It is a precious source of fiber, iron, folate, and vitamin C. Moreover, consuming food high in dietary fiber has several valuable functions, such as maintaining good health and a healthy digestive system. Lettuce is also a decent source of several health-beneficial bioactive metabolites [11]. Red lettuce has a higher phenolic content and has more antioxidant characteristics than green lettuce owing to its higher abundance of anthocyanins [12,13]. The sesquiterpene lactones present in lettuce are responsible for its bitter taste; they are also believed to contribute to the plants’ defense against herbivores. Various pharmacological activities have been attributed to sesquiterpene lactones, leading to promising therapeutic applications [14]. Many sesquiterpene lactones including those with oxalate and sulfate conjugates have been reported in lettuce [15].

Several phenolic metabolites have been isolated from iceberg lettuce such as caffeoyl derivatives and flavonoids, especially quercetin and luteolin glycosides [16]. Anthocyanins, which are a flavonoid subgroup, are responsible for the characteristic red color of red leaf lettuce. Consequently, red leaf lettuce showed a better antioxidant capacity, which has a potential impact on human health [13].

It is beneficial to investigate the effect of different fertilization regimes on an easy-to-use hydroponic system for providing proper support to enhance the nutritional quality of the most popular vegetables, including lettuce. There is a consensus that the provision of nutrients at a sufficient level has a significant impact on crop yield. Consequently, a reduction in mineral elements might affect plant metabolic processes and subsequently result in reduced crop quality. The supplementation of crops with minerals and vitamins is one of the most effective strategies to improve plant and human health [17,18]. The aim of this study, hence, is to emphasize the importance of S nutrition to different lettuce cultivars and to investigate its influence on the plant growth, quality, and biosynthesis of beneficial secondary metabolites with biological activities. Owing to the important role of minerals in human health, the study highlights the diversity of crosstalk between S and other mineral nutrients in lettuce plants. We followed the hypotheses: the antibacterial activity of lettuce heads will be induced by sulfur treatment. Moreover, S will boost crop quality in addition to plants and human health.

## 2. Materials and Methods

### 2.1. Plant Materials and Growth Conditions

Three different lettuce cultivars, namely, Pazmanea RZ (butterhead, green, V1), Hawking RZ (green multi-leaf lettuce, V2), and Barlach RZ (red multi-leaf, V3) were selected owing to their different quality characteristics. Seeds were sterilized and homogenously spread into sandwich blots and placed in a container of water. Well-grown and 14 day-old seedlings were then transferred into a 10 L randomly arranged individual darkened containers, which were kept under standard greenhouse conditions with a day/night cycle of 18/14 °C and a 14 h photoperiod. There were four replicates per cultivar arranged in a completely randomized design. The nutrient solution used was composed of (g L^−1^) the following nutrients: Macronutrient (mM) KNO_3_ = 2, NH_4_H_2_PO_4_ = 0.5; Ca(NO_3_)_2_.4H_2_O = 2, Micronutrient (µM) Fe-EDTA = 60, H_3_BO_3_ = 2.98, MnSO_4_.H_2_O = 2.0, ZnSO_4_.7H_2_O = 0.62, CuSO_4_.6H_2_O = 0.31, (NH_4_)_2_Mo_7_O_24_ = 0.1 [19]. The pH was maintained between pH 5.5 and 6.5. Two S-treatments (0.5 mM) with either MgSO_4_.7H_2_O (+S) or MgCl_2_.6H_2_O (−S) were performed by regular replacement of the nutrient solution, which was replaced every week until harvesting on day 56. Subsequently, the fresh weight (FW) and number of leaves were recorded immediately after the plants were harvested. The lettuce leaves were frozen with liquid nitrogen and immediately placed in a freeze-dryer (Gamma1–20, Christ, Osterode am Harz, Germany) at −53 °C (72 h) to determine the dry weight (DW). The dried samples were then ground to a fine powder for further analyses. Lettuce head powder were extracted with methanol (500 mL). The organic solvent was evaporated to dryness in a rotary evaporator. The crude extracts were stored in the dark at −20 °C prior to chemical profiling and bioactivity analyses.

### 2.2. Determination of Inorganic Anions, Organic Acids and Water-Soluble Sugars

Anions including chloride, nitrate, sulfate, and phosphate in addition to organic acids (OAs) and water-soluble sugar (WSS) concentrations were determined by ion-chromatography (IC-5000 Dionex/ Thermo Scientific, Waltham, MA, USA). The extraction of WSS and free inorganic anions was performed following the procedure reported by [20] with slight modifications. Dried and powdered lettuce leaves were boiled in 1.5 mL of sterile deionized water for 5 minutes, mixed thoroughly by vortex, and immediately incubated in an ice-water bath for half an hour. Subsequently, the mixtures were centrifuged. Moreover, proteins were excluded from the supernatant by chloroform extraction. The supernatant was filtered on strata C18-column (Phenomenex, Torrance, CA, USA) prior to ion-chromatography.

### 2.3. Mineral Analyses Using ICP-MS

Dried and finely ground samples were used to determine the mineral profile analysis, which was carried out by inductively coupled plasma mass spectrometry (ICP-MS; Agilent Technologies 7700 Series, Böblingen, Germany) in accordance with DIN EN ISO 17294-2 (Deutsches Institut für Normung, Berlin, Germany, 2005). For elemental determination, freeze-dried lettuce leaves (200 mg) were digested with 10 mL of 69% HNO_3_ (ROTIPURAN^®^ Supra for ICP, 69%) by using a closed-vessels microwave digestion system (MARS 6 Xpress, CEM Corporation, Matthews, NC, USA) under the following conditions: 2 min at 100 °C, 1 min at 120 °C, 20 min at 180 °C and 20 min cooling time. Subsequently, the digested lettuce samples were diluted with Milli-Q water (18.2 MΩ cm conductivity) to 100 mL and stored at 4 °C until further analysis. The concentrations of the macronutrients (magnesium, Mg^2+^; phosphorus, P; potassium, K^+^ and calcium, Ca^2+^) and micronutrients (iron, Fe; manganese, Mn; Copper, Cu; zinc, Zn) were determined by ICP-MS as described by [21].

### 2.4. Determination of Sulfur and Nitrogen Concentrations Using an Elemental Analyzer

The sulfur and nitrogen concentrations of the lettuce plants were determined using 5 ± 0.1 mg of freeze-dried samples, which was combined with 5–10 mg Wolfram-VI-oxide for optimal combustion. Measurement of S and N concentrations was performed by using an elemental analyzer (Flash EA1112, Thermo Fisher Scientific, Milano, Italy), as described previously [22].

### 2.5. LC-DAD-MS Analyses

The crude extracts of four biological replicates for each treatment were analyzed with an VWR HPLC system equipped with a Diode Array Detector L-2450 (DAD) and an Esquire 4000 Bruker Daltonics mass spectrometer (ESIMS). Methanolic solutions of the crude extracts (40 mg/mL) were injected twice (10 μL × 2) into the HPLC-DAD-MS system after filtering through a 0.2 μm PTFE syringe filter. A monolithic C18 column (Onyx monolithic C18, 100 × 3.0 mm, Phenomenex, Torrance, CA, USA) was used for the chromatographic separations at 40 °C. The mobile phase used was A: MilliQ-water/0.1% formic acid (Fluka) and B: Acetonitrile (Actu-All Chemicals BV)/0.1% formic acid; pumped at a rate of 1.0 mL/min with a gradient of B/A: 1:99 to 60:40, 0–10 min, 60:40 to 100:0, 10–12 min, and a column washing and reconditioning phase until 19 min. The MS and MS/MS (MS^2^) data were acquired for all samples at positive and ionization mode. The MS^2^ data of all the samples were uploaded to the publicly available Global Natural Product Social molecular networking (GNPS) platform (https://gnps.ucsd.edu/ProteoSAFe/static/gnps-splash.jsp (accessed on 21 October 2020)) for an automatic dereplication of the known compounds. A manual dereplication was also conducted by analyzing the MS/MS patterns, UV absorption, and comparison with published data. The Competitive Fragmentation Modeling for Metabolite Identification (CFM-ID) platform (http://cfmid.wishartlab.com (accessed on 21 October 2020)) was used to predict the fragments of a molecule. The open-access online databases ReSpect (RIKEN MS^n^ spectral database for phytochemicals) and MoNA (MassBank of North America) were used to search for the MS^2^ fragmentation patterns of the known molecules.

### 2.6. Antibacterial Assay

An antibacterial assay was performed using a *Staphylococcus aureus* DSM 346 strain. The cultivation took place in TSB medium (1.2% tryptic soy broth; 0.5% NaCl). Overnight culture of the test organism was prepared and diluted to an optical density (600 nm) of 0.01. The samples (20 mg/mL stock solution) were dissolved in the medium and transferred into a 96-well microtiter plate and 200 µL of the diluted culture was added to each well. The inoculated microplate was incubated for 5 h at 37 °C and 200 rpm. For detection of the inhibitory effect of the samples, 10 µL of a resazurin solution (0.3 mg/mL phosphate-buffered saline) was added to each well and left for incubation for 5 min. The fluorescence signal (560 nm/590 nm) was measured using a microplate reader (Tecan Infinite M200, Männedorf, Switzerland). Chloramphenicol was used as a positive control.

### 2.7. DPPH Radical Scavenging Activity

The capability of the lettuce leaves extracts to act as antioxidants was determined using the free radical 2,2-diphenyl-1-picrylhydrazin (DPPH). The assay was performed by dissolving the DPPH in methanol to a final concentration of 200 µM. Extracts were also dissolved in methanol and pipetted (100 µL) into a 96 well microplate. The reaction was started with the addition of 100 µL DPPH solution. After 30 min of incubation in the dark at room temperature, the radical scavenging activity of the extracts was measured at 517 nm using a microplate reader (Tecan Infinite M200, Tecan, Crailsheim, Germany). Ascorbic acid was used as a positive control.

### 2.8. Statistical Analyses

MS data of all samples were processed with the open-access software MZmine (version 2.53, Okinawa, Japan). The peak area, which is linearly correlated with the quantity of the features, is calculated for each detected feature. Background noise and solvent blank were extracted from samples. Processed data obtained in positive and negative ionization modes were combined for different cultivars separately via an R package named MSCombine. The combined MS data were uploaded to the online platform MetaboAnalyst (www.metaboanalyst.ca (accessed on 3 November 2020) for comprehensive metabolomic study and statistical analysis. Data of growth, primary metabolites and elemental composition were statistically analyzed using two-way (treatment × cultivar) analysis of variance ANOVA. Significant differences among the means were determined by Tukey’s HSD test (*p* ≤ 0.05).

## 3. Results and Discussion

### 3.1. Plant Biomass

**Figure 1:**
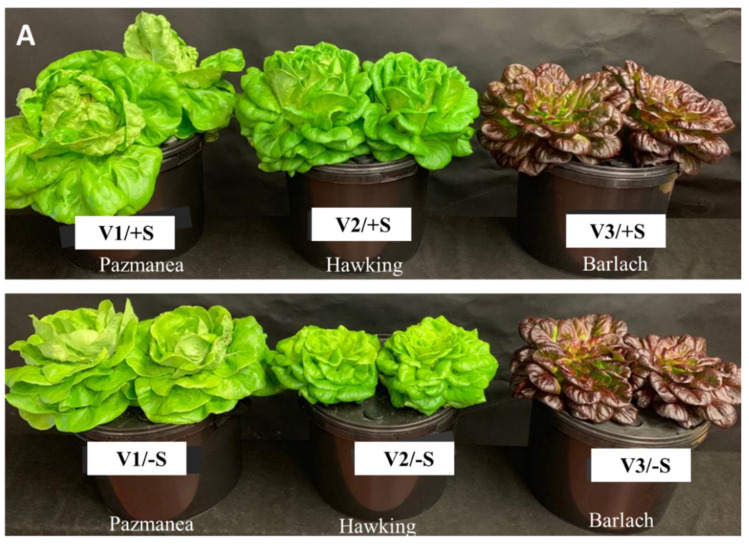

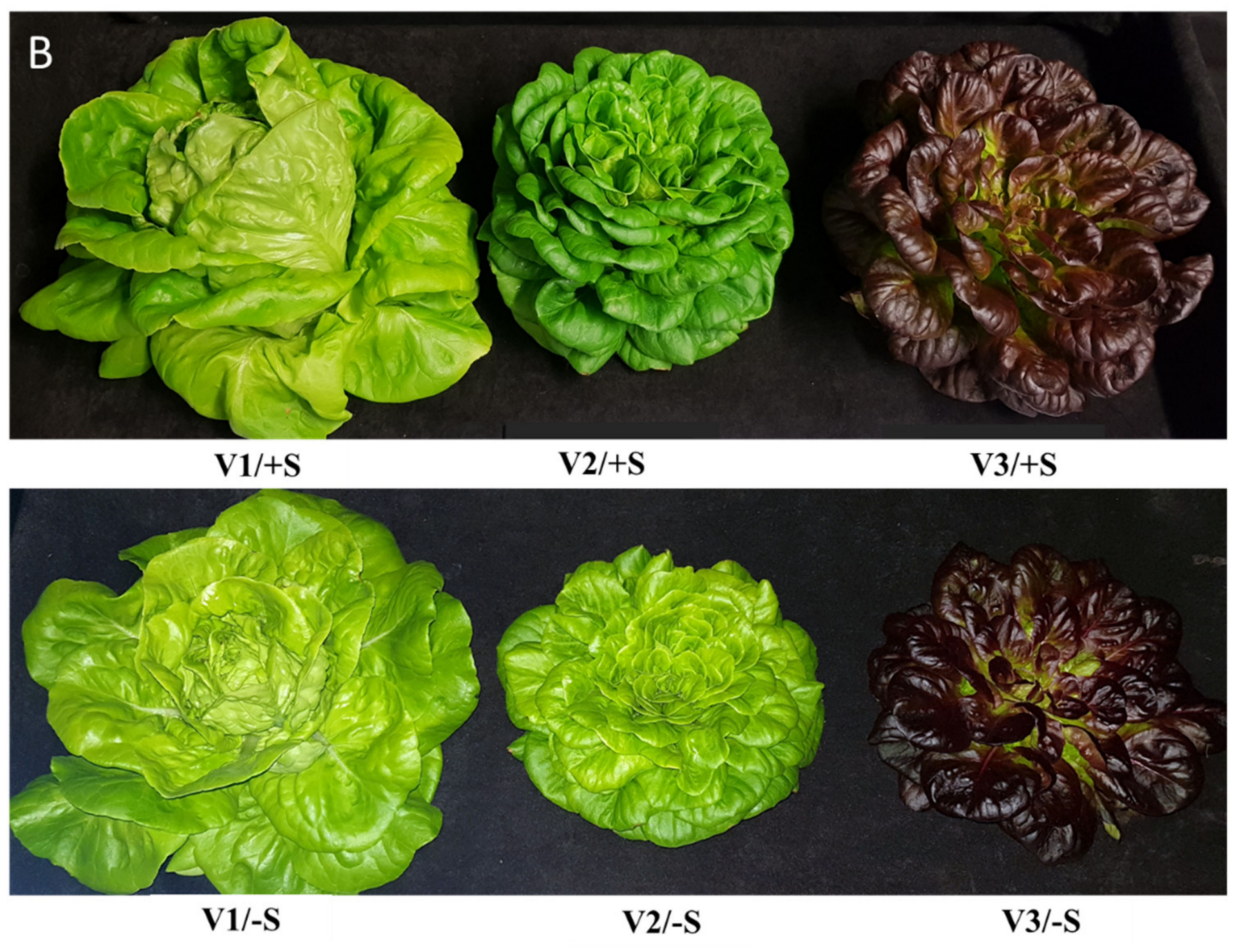
Biomass productions in three lettuce cultivars, namely, Pazmanea RZ (green butterhead, V1), Hawking RZ (green multi-leaf lettuce, V2), and Barlach RZ (red multi-leaf, V3) grown in hydroponic system and treated with magnesium sulfate (+S) or magnesium chloride (−S) at a final concentration of 0.5 mM. (**A**): Side view (**B**): Top view of the lettuce heads.

**Figure 2:**
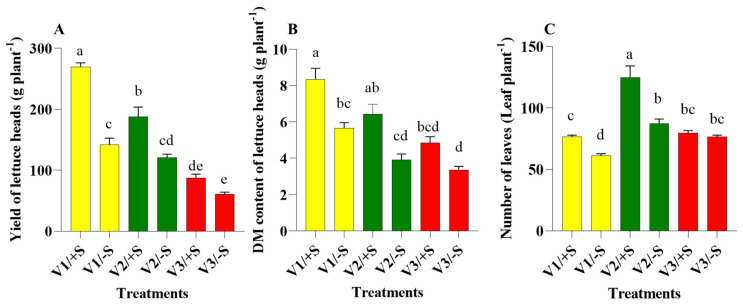
(**A**): Yield, (**B**): dry matter (DM) accumulation, and (**C**): number of leaves in three lettuce cultivars; namely, Pazmanea RZ (green butterhead, V1), Hawking RZ (green multi-leaf lettuce, V2), and Barlach RZ (red multi-leaf, V3) grown in hydroponic system and treated with magnesium sulfate (+S) or magnesium chloride (−S) at a final concentration of 0.5 mM. Data presented are the means ± SDs of four replicates. Different letters show statistically significant differences among all the treatments (*p* ≤ 0.05; Tukey’s test).

The current findings showed a remarkable variation in terms of plant biomass production between the three examined cultivars. Data reported here indicates that S provision had a significant influence on the final yield and dry matter (DM) accumulation of V1 and V2 cultivars (Figure 1 and Figure 2). Under +S conditions, yield and total-plant DM of V1 were significantly higher than that of V2 and V3, suggesting that the V1 performed better than the other two cultivars. Contrary to S sufficiency, S depletion resulted in significantly greater reductions in the yield and DM of V1 and V2 plants than in V3 plants (−47% vs. −36% vs. −29% for yield and −32% vs. −39% vs. −29% for DM, respectively) (Figure 2). S deprivation is considered to be a constraint to yield in various crop production systems globally [23]. Although relatively acclimated to S starvation, however, the V3 cultivar revealed the least amount of head yield under both +S and −S conditions (Figure 1 and Figure 2A).

Apart from the yield and DM, the number of leaves in V1 and V2 exhibited strikingly higher levels of sensitivity to S depletion than the V3 (Figure 1 and Figure 2C). Specifically, the V3 cultivar was negatively affected to a lower extent (reduction by 4%) than in V1 (reduction by 34%) and V2 (reduction by 30%). Collectively, our findings support the observation that the red V3 cultivar was more optimally acclimatized to cope with −S than the green cultivars (Figure 1 and Figure 2).

Owing to the role of S as an essential macronutrient for plant growth and metabolism, the negative effects observed in crop growth and yield in response to S deficiency have been reported [23,24,25,26]. Therefore, the addition of S is necessary for the yield and DM of lettuce to be increased [27]. This is supported, for instance, by the higher yield of the green cultivar V1 under the +S level. On the other hand, the red-V3 cultivar did not show any significant differences regarding yield, DM, and the number of leaves under +S and −S conditions. These findings were in agreement with the results reported by Senizza et al. [28], who indicated that the red cultivar was much more responsive to nutritional deprivation, compared with that of the green cultivar [27]. Thus, the V3 cultivar could be used as a good platform to investigate the plant acclimation to S-deprivation conditions. Our results represent a good foundation for further comparison of these cultivars at molecular levels, which enables us to understand the genetic network involved in the regulation of lettuce responses to S deprivation.

### 3.2. Free Inorganic Anions, WSS, and OAs

In light of its important characteristics as an essential macronutrient in plant biological processes, S deficiency causes an internal reprogramming of primary and secondary metabolism, which alters the concentrations of distinct metabolites in S-deprived plants. In consideration of these complicated issues, the levels of various inorganic anions (i.e., Cl^−^, SO_4_^2−^, NO_3_^−^ and PO_4_^3−^), coupled with some primary metabolites including WSS and OAs in the three cultivars, were precisely determined under +S and −S conditions. These organic and inorganic constituents can substantially contribute to improving the crop-quality attributes relevant to human nutrition. Based on this study, the concentration of Cl^−^ was significantly increased in plants treated with MgCl_2_, whereas the Cl^−^ level in plants subjected to +S was significantly reduced (Table 1). Despite the remarkable increment of Cl^−^ in plants treated with −S, the concentrations reached by this nutrient remain relatively low (<15 mg g^−1^ DM in the three cultivars) and there are no visual toxicity signs (Figure 1). More recent studies have shown that an optimal concentration of Cl^−^ ranges from 2 to 20 mg g^−1^ DM, which is classified as a typical level of a macronutrient [29]. However, these results are not aligned with the premise that lettuce is very prone to Cl^−^ toxicity. Building on the previous work, lettuce has been categorized among the chlorophobic crops, which is more likely to prompt a better response to sulfate- than chlorine-based fertilizers [30]. As one might expect, SO_4_^2−^ concentrations were significantly enhanced in +S plants (Table 1). This S enrichment could have an important implication on the final quality parameters of lettuce plants, as explained below.

NO_3_^−^ concentration was significantly enhanced in the green V2 cultivar under S depletion (by 140%) (Table 1). Previous reports have indicated that NO_3_^−^ levels can be increased by lowering the S fertilization [26,31]. High NO_3_^−^ intake from leafy vegetables or drinking water is associated with negative effects on human health [32]. Leafy vegetables are considered as the substantial source of dietary NO_3_^−^ intake in particular, owing to their NO_3_^−^ accumulation capacity. In this regard, a lower NO_3_^−^ content in edible vegetable crops has significant health benefits likely due to the conversion to nitrites, which can interact with the amino acid residues of hemoglobin and subsequently enables the adequate transport of blood oxygen [33]. Alternatively, NO_3_^−^ may react with secondary amines to form nitrosamines, which are associated with an increased risk of gastrointestinal cancer [34]. In many ways, S nutrition plays a crucial role in NO_3_^−^ uptake and the activities of N assimilatory enzymes. In a similar manner to NO_3_^−^, PO_4_^3−^ concentration was significantly increased in V2 cultivar under −S (by 53%), whereas V1 and V3 remained unaffected (Table 1). Excessive PO_4_^3−^ levels in blood serum might cause some medical complications in healthy individuals, such as renal impairment and cardiovascular diseases [35]. Accordingly, S supply could indirectly contribute to improving the quality of lettuce by lowering the excessive levels of NO_3_^−^ and PO_4_^3−^ in the harvested products.

WSS contributes substantially to defining the crispness and sweetness of lettuce [36]. Among these soluble sugars, glucose concentration was significantly diminished in S-deficient V1 (by 57%) and V2 (by 61%) plants, but exhibited a slight tendency for increment in V3 plants (increased by 14%) under similar conditions (Table 1). In contrast to glucose, fructose concentration was not significantly affected by −S (Table 1). With the exception of V3, which remained unaffected (decreased by 15%), the concentration of sucrose was significantly declined in both green cultivars in response to S-limiting conditions (decreased by 43% and 45% in V1 and V2, respectively) (Table 1). These results align with a previous report that indicated that lower-than-normal WSS concentrations were measured in S-deficient plants [37]. Apart from WSS, the concentration of OAs showed remarkable variations (Table 1). While the concentration of malic acid remained unaffected in V3 plants (decreased by 1%), the concentration of this phytochemical was significantly decreased in V1 (by 27%) and V2 (by 21%) cultivars under −S treatment. The sensitivity of malic acid to S deprivation was previously documented in *Arabidopsis* plants [38]. In contrast, the concentrations of oxalic acid and citric acid remained unchanged in −S plants of the three tested cultivars (Table 1). Together with WSS, OAs are well known to possess a major effect on taste, being responsible for sourness and contributing to the flavor [39]. Collectively, the present results indicate that the sufficient amounts of S are critical in promoting the level of WSS and OAs, both of which are the main drivers in maintaining nutritional value and quality.

### 3.3. Elemental Composition

The mineral profile (micro and macro) in the three lettuce cultivars is depicted in Table 2. Selimination resulted in a significant decrease in K^+^ level in the heads of V1 (by 25%) and V2 (by 18%) plants, while V3 showed a better acclimation to −S (decreased only by 7%). The interactions of SO_4_^2−^ with other nutrients, including K^+^, were studied by Reich et al. using Chinese cabbage as a test plant [40]. It was found that the concentration of K^+^ was positively correlated with the shoot-S content. The authors concluded that K^+^ as a counter cation for SO_4_^2−^ via xylem loading and storage in leaves vacuoles might play an important role in plant growth and acclimation. Additionally, it was previously shown that K^+^ plays an important role in defining the color, texture, and sweetness in muskmelons [41]. Apart from K^+^, the concentrations of Ca^2+^, Mg^2+^, and total *p* remained unaffected in all three cultivars under S deprivation (Table 2).

Contrary to the above-mentioned nutrients, the total N concentration was significantly enhanced in the green cultivars (by 12% and 14% in V1 and V2, respectively), whereas the N level remained unchanged in the red V3 cultivar under low S (Table 2). S is important for N assimilation in plants. For instance, it has been reported that adequate-S fertilization is required for wheat to efficiently utilize N for protein biosynthesis and to reach a full potential yield [42]. Thus, S starvation may result in significant changes in N metabolism including a decrease in protein synthesis and accumulation of non-proteinogenic N [40]. As suspected, total S was significantly decreased in plants including the relatively acclimated V3 under S deprivation (Table 2). Consequently, a significant increase in the N/S ratio was observed in all cultivars under S deprivation (Table 2). The N/S ratio represents one of the most important indicators of S limitation. Several authors have reported a significant increase in N/S ratios in sugar beet and alfalfa under similar conditions [43,44].

As for the micronutrients, the current study revealed a significant decrease in Mn concentrations in the three cultivars under −S (Table 2). It has been reported that Mn acquisition can be affected by S supply and S application can promote Mn uptake at the cellular level [45]. Given the close synchronization with S, it is likely that the decrease in Mn concentrations observed could be attributed to S scarcity. Following the exposure to −S, Fe concentration was significantly reduced, especially in the V1 cultivar (Table 2). This result is supported by Zuchi et al. who observed a significant Fe decrease in S-deprived tomato plants [46]. While Cu levels remained unaltered among the three cultivars, the concentration of Zn was significantly increased in V2 under S limitation (Table 2). This response was also supported by a previous study examining the impact of S deficiency [40]. Putting it simply, lettuce is a rich source of macro- and micronutrients; thus, adequate-S supply is necessary to improve the contents of these nutrients in the final harvested parts (i.e., quality aspects).

### 3.4. Comparative Secondary Metabolite Profile of the Three Lettuce Cultivars under S-Sufficient (+S) and S-Deficient (−S) Conditions

Annotation of small molecules affected by different growth conditions by metabolomics provides an overview of the physiological processes demonstrated under particular stress conditions [47]. In our experimental approach, untargeted LC-DAD-MS-based metabolomics were used to investigate differences in the metabolite profile of the leaves of three lettuce cultivars. In-depth analyses of the UV absorption and MS/MS fragmentation patterns led to the identification of 16 known secondary molecules from all three cultivars (Table 3). Statistical analyses (PCA, fold change, *t*-test, and volcano plot) were performed separately to assess the metabolomic variations among all three cultivars under both +S and −S conditions.

**Figure 3:**
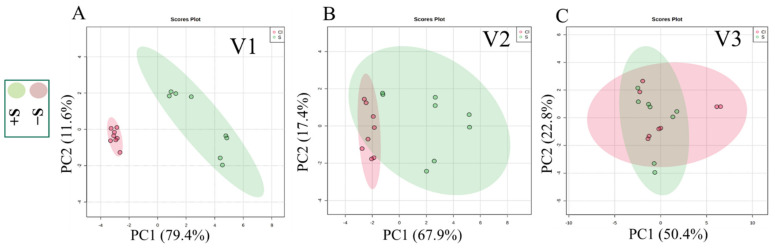
Principal component analysis (PCA) scores plot of the three lettuce cultivars under both +S and −S conditions. conditions; (**A**): V1, (**B**): V2, (**C**): V3.

The PCA analysis of V1 separated cultivars under +S and −S without any overlapping, wherein the first two principal components accounted for 91.0% of the total variance (Figure 3), and revealed significant differences between metabolites present in V1 under +S and −S conditions. A similar result was obtained in the PCA analysis of V2. Despite the observation of some overlaps, notable differences in the composition of secondary metabolites were obvious between the two growth conditions. Regarding V3, PCA analysis did not separate the cultivars under +S and −S conditions, although the first two principal components accounted for 73.2% of the total variance (Figure 3), suggesting little difference in the production of metabolites under +S and −S conditions.

**Figure 4:**
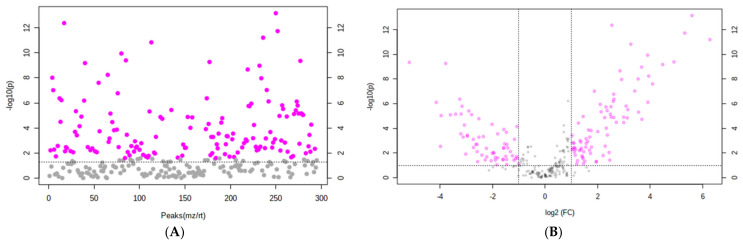
*t*-test and volcano plot analysis of the three lettuce cultivars under +S and −S conditions. (**A**): *t*-test, pink nodes represent important features selected by *t*-tests with threshold (*p*-value) 0.05. (**B**): volcano plot analysis, pink circles represent metabolites selected by volcano plot with fold change threshold (−S/+S) 2 and *t*-tests threshold 0.1. Both fold changes and *p*-values are log-transformed.

Additional methods, including a *t*-test and volcano plots, were used to further explore the differences of secondary metabolites of cultivars under both +S and −S conditions. Important features were selected by a *t*-test with a threshold (*p* < 0.05) level (Figure 4A). Metabolites with a fold change (FC) (−S/+S) > 2 and *p*-value (*t*-test) < 0.1 were included in the volcano plot (Figure 4B). Fold change and *p*-values (*t*-test) of metabolites identified in V1 and V2 are shown in Table 4.

**Figure 5:**
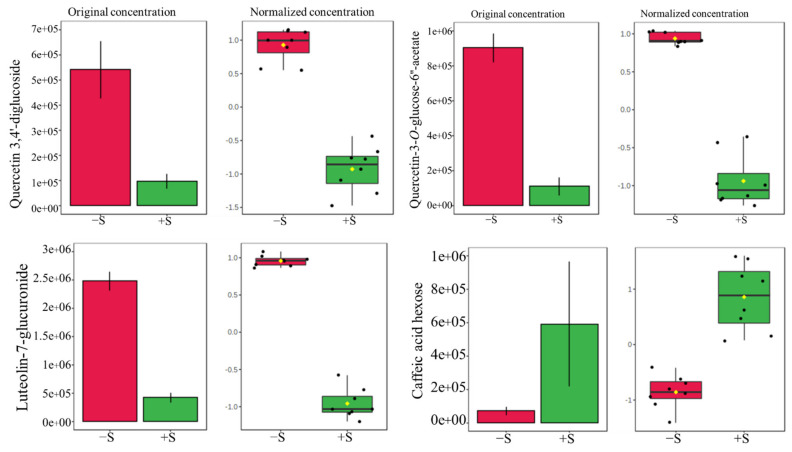

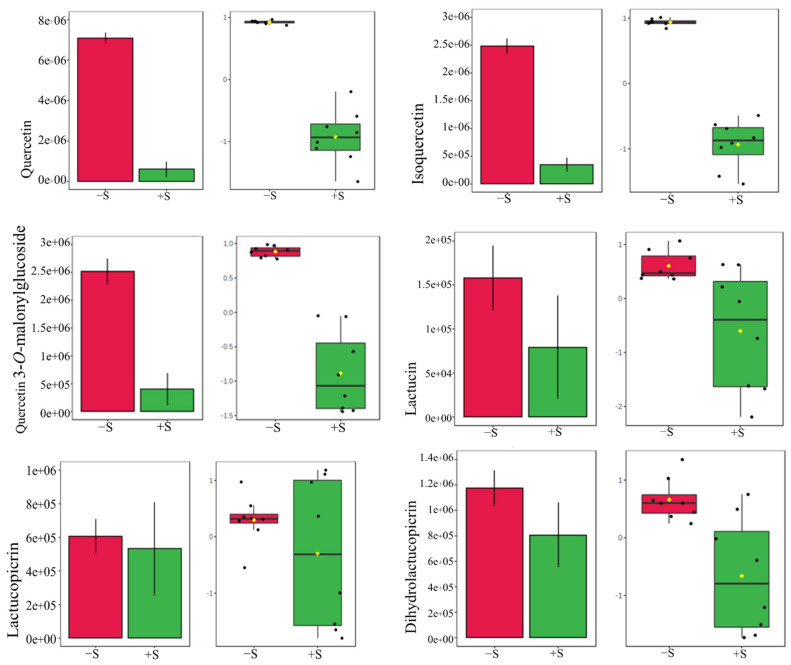
Detailed quantitative analysis of compounds detected in butterhead green lettuce (V1) grown under +S and −S conditions.

In the V1 cultivar, flavonoids and flavonoid glycosides—namely, quercetin 3,4′-diglucoside (FC 30.015), quercetin-3-*O*-glucose-6″-acetate (FC 12.783), quercetin (FC 11.674), isoquercetin (FC 7.0765), quercetin 3-*O*-malonylglucoside (FC 6.5456), and luteolin-7-glucuronide (FC 5.8241)—showed up in much higher quantities under −S conditions compared to those detected in V1 under the +S level, as suggested by the large FC (−S/+S) and small *p*-values (Table 4). In contrast, caffeic acid hexose (FC 0.056472) was greater in plants under the +S condition (Table 4, Figure 5). No significant difference in the abundance of dicaffeoyltartaric acid, dicaffeoylquinic acid or the sesquiterpene lactones dihydrolactucopicrin, lactucin and lactucopicrin was observed under +S or −S conditions.

Our data agreed with the premise that flavonoids of different chemical subclasses increased in S-deficient plants, as indicated by [38]. Additionally, Zhang et al. (2011) studied the metabolite profiling of *Arabidopsis* seedlings under exogenous sinalbin and sulfur starvation conditions; the authors found that all flavonol glycosides and sinapinic acid esters were produced in higher amounts in S-deprived plants compared with control plants [48]. Nikiforova et al. (2003) found that genes involved in flavonoid, auxin, and jasmonate biosynthetic pathways in *Arabidopsis thaliana* were upregulated under sulfur depletion [49].

To mitigate the adverse effects of different abiotic stresses, plants have mechanisms that are coordinated throughout the plant tissues in a systemic manner through chemical signals [50], although, not all mechanisms of plant responses to different stress conditions are completely known. However, flavonoids constitute a metabolic pathway for plants adaptation, which enhances the resistance against biotic and abiotic stress [51]. This can explain the observed accumulation of flavonoids in V1 plants under −S conditions.

The FC (−S/+S) and their according Log2 values indicated fewer significant changes for V2 plants grown under +S and −S conditions (Table 4). Twelve metabolites were detected in V2, including flavonoids and flavonoids glycosides (Table 4). Under −S conditions, the abundance of isoquercetin, quercetin, 5-CQA, and quercetin-3,4′-diglucoside were increased approximately 2-fold (Table 4, Figure S1). This is in agreement with a previous study on *Apocynum venetum* that showed enhancement of the amounts of isoquercetin and other flavonoids under salt stress [60]. However, no significant differences were observed in the amount of other metabolites annotated in V2 (Table 4, Figure S1).

The metabolites cyanidin-3-*O*-galactoside and kaempferol malonylglucoside were found exclusively in V3 and were not detected in any other cultivars (V1 and V2). Their levels remained unchanged in all plants under +S and −S conditions (Figure S2). Moreover, no significant changes were observed in the amount of other phenolic compounds such as quercetin 3-*O*-malonylglucoside, quercetin, isoquercetin, quercetin 3,4′-diglucoside, quercetin-3-*O*-glucose-6′′-acetate, quercetin 3′,4′-di-*O*-β-d-glucopyranoside, caffeic acid hexose, luteolin-7-glucuronide, 5-CQA, DCTA, DCQA, dihydrolactucopicrin, and lactucopicrin (Figure S2).

Several phenolic metabolites have been identified in red leaf lettuce, including glycosides of cyanidin, quercetin, and luteolin, in addition to caffeic acid and their derivatives [61]. The major difference between red and green lettuce was the presence of anthocyanins in the red lettuce cultivar. The cyanidin glycoside derivatives reflect the quality of the red lettuce as they are responsible for the red color of the leaves [62].

### 3.5. Antibacterial Activity

Sulfur is known as a potential element that protects plants and animals against parasites. Weld and Gunther (1947) [63] indicated that saturated solutions of S in alcohol and in carbowax (when diluted with broth) are highly active against gram-positive bacteria [64]. Cysteine has been found to boost the effectiveness of various bactericidal antibiotics against Gram-negative bacterial persisters by a combined mechanism, including the enhancement of bacterial respiration to produce toxic reactive oxygen species (ROS) and, subsequently, converting persister cells to metabolically active cells [65]. Moreover, S-containing compounds such as sulforaphene have a wide range and remarkable antibacterial properties against drug-resistant strains including *Helicobacter pylori* and *S. aureus* [66]. In the current study, two cultivars treated with S showed moderate antibacterial activity against *S. aureus*; both V1 and V2 exhibited activity of 39.06 ± 7.8% at 200 µg/mL concentrations. Both cultivars did not show any antibacterial activity under −S conditions (Table 5). The red multi-leaf V3 lettuce, grown under both +S and −S conditions, was equally active, inhibiting the growth of *S. aureus* 44.34 ± 5.41% and 43.55 ± 7.34%, respectively, at the same test concentration (Table 5).

The potential antibacterial properties could be due to the synergistic interactions between phenolic metabolites and other compounds in plant extracts; understanding this interplay would allow researchers to develop an alternative tactic for the use of antibiotics against multidrug-resistant bacteria. Furthermore, cafeic acid hexose was present in V1, at a higher concentrations in V1 grown under +S level than in V1 plants grown under −S condition (Table 4 and Figure 4). The antibacterial activity of cafeic acid has been reported in several studies [67,68,69].

### 3.6. Free radical Scavanging (Antioxidant) Assay

Antioxidants are compounds that protect cells from damage by scavenging and neutralizing free radicals. Nutrient-rich plant foods, including fruits and vegetables, are important sources of antioxidants, which can reduce the risk of chronic oxidative-stress-related diseases [70]. The antioxidant activity of plant extracts primarily stems from phytochemicals including polyphenols, flavonoids, anthocyanins, carotenoids and vitamins E and C [71]. Accordingly, determination of the antioxidant potential remains a good approach, particularly to discover interesting sources of natural antioxidants that can be used to formulate functional foods and/or nutraceuticals [72]. A DPPH assay is an accepted method and is widely used in plant biochemistry to evaluate the radical scavenging activity of extracts.

In the present study, the V3 lettuce plants showed promising radical scavenging (antioxidant) activity against DPPH radical with IC_50_ values of 60.5 ± 0.06 µg/mL (under +S) and 62.8 ± 1.29 µg/mL (under −S), respectively. Ascorbic acid (positive control) showed the highest antioxidant activity (4.1 ± 0.0 µg/mL). The V1 and V2 lettuce cultivars were not active even at the highest test concentrations (IC_50_ > 200 µg/mL) under both +S and −S conditions (Table 5). The significantly higher antioxidant activity of red lettuce in comparison with the green ones might be linked to the presence of anthocyanins, especially cyanidin 3-*O*-galactoside, which was only detected in V3 lettuce plants under +S and −S levels.

Anthocyanins are well known as potential antioxidants. Owing to the presence of phenolic hydroxyl moieties in anthocyanins, which act as hydrogen donors, they are able to capture free radicals and stop ROS-induced damage. Moreover, the ortho-diphenolic hydroxyl group of anthocyanins can bind metal ions and prevent harmful consequences by inhibiting the catalytic effects of metal ions on oxidation reaction [73]. It is very important to note that cyanidin-3-*O*-galactoside demonstrated superior antioxidant capacity in different antioxidant assays including ORAC, DPPH, ABTS, FRAP, and O^2^, in comparison to synthetic antioxidants BHT, BHA, and Trolox, in addition to anti-inflammatory, anti-diabetic, and cytoprotective activities [74,75]. Besides human health benefits, anthocyanins have an important role in maintaining the health of plants and protecting them from environmental and physiological stresses [76].

## 4. Conclusions

Lettuce is one of the most important dietary vegetables consumed all over the world. Hence, it remains highly important to improve its nutritional constituents that nourish human health. The analyses presented here indicate that S application can promote plant production, the overall quality regarding the elemental composition, and primary and secondary metabolites, especially of the green lettuce cultivars (V1 and V2). Our results show that the red V3 is a precious source of beneficial polyphenolic compounds that has a better S-stress acclimation than the other green cultivars. Owing to its anthocyanin content, it possesses a promising radical scavenging potential. The novelty of this work lies in the capability to enhance the antibacterial properties of green lettuce cultivars via the judicious application of S. Further work needs to be done to explore the effects of different S regimes on the antibacterial activity and to identify the main compounds involved.

## Figures and Tables

**Table 1:** Inorganic anions and primary metabolites (mg g^−1^ DM) in three lettuce cultivars, namely, Pazmanea RZ (green butterhead, V1), Hawking RZ (green multi-leaf lettuce, V2), and Barlach RZ (red multi-leaf, V3) grown in hydroponic system and treated with magnesium sulfate (S-sufficient) or magnesium chloride (S-deletion) at a final concentration of 0.5 mM.

Treatments						
	V1/+S	V1/−S	V2/+S	V2/−S	V3/+S	V3/−S
Anions						
Cl^−^	0.47 ± 0.08 b	13.4 ± 0.96 a	0.49 ± 0.06 b	16.2 ± 1.12 a	0.57 ± 0.02 b	14.9 ± 0.41 a
SO_4_^2−^	1.35 ± 0.07 a	0.21 ± 0.03 b	1.42 ± 0.17 a	0.26 ± 0.03 b	1.33 ± 0.18 a	0.54 ± 0.13 b
NO_3_^−^	0.84 ± 0.17 b	1.35 ± 0.31 b	2.36 ± 0.50 b	5.67 ± 0.28 a	1.65 ± 0.54 b	1.78 ± 0.28 b
PO_4_^3−^	3.86 ± 0.15 b	5.58 ± 0.62 ab	4.41 ± 0.30 b	6.75 ± 0.47 a	5.30 ± 0.27 ab	4.99 ± 0.28 ab
**Soluble sugars**						
Glucose	5.07 ± 0.22 b	2.14 ± 0.27 c	4.59 ± 0.39 b	1.73 ± 0.15 c	7.29 ± 0.17 a	8.37 ± 0.62 a
Fructose	5.85 ± 0.09 a	5.66 ± 0.39 a	3.66 ± 0.492 a	3.58 ± 0.34 a	4.15 ± 0.20 a	4.03 ± 0.33 a
Sucrose	4.39 ± 0.39 a	2.50± 0.21 bc	4.40 ± 0.31 ab	2.41 ± 0.16 c	4.52± 0.37 a	3.84 ± 0.21 abc
**Organic acids**						
Malic acid	13.82 ± 0.49 a	10.09 ± 0.5 b	14.47 ± 0.58 a	11.37 ± 0.18 b	9.74 ± 0.37 b	9.628 ± 0.516 b
Oxalic acid	1.62 ± 0.76 a	1.30 ± 0.04 ab	1.67 ± 0.13 a	1.21 ± 0.07 ab	1.41 ± 0.04 ab	1.16 ± 0.10 b
Citric acid	1.47 ± 0.13 a	1.30 ± 0.10 a	1.39 ± 0.12 a	1.35 ± 0.17 a	1.09 ± 0.07 a	1.19 ± 0.10 a

Data presented are the means ± SDs of four replicates. Different letters show statistically significant differences among all the treatments (*p* ≤ 0.05; Tukey’s test).

**Table 2:** Macronutrient (mg g^−1^ DM) and micronutrient (μg g^−1^ DM) concentrations in three lettuce cultivars; namely, Pazmanea RZ (green butterhead, V1), Hawking RZ (green multi-leaf lettuce, V2), and Barlach RZ (red multi-leaf, V3) grown in a hydroponic system and treated with magnesium sulfate (+S) or magnesium chloride (−S) at a final concentration of 0.5 mM.

Treatments						
	V1/+S	V1/−S	V2/+S	V2/−S	V3/+S	V3/−S
**Macronutrients**						
P	8.88 ± 0.22 bc	9.01 ± 0.29 b	13.0 ± 0.06 a	12.5 ± 0.24 a	7.76 ± 0.12 cd	7.08 ± 0.18 d
Mg	3.38 ± 0.21 b	2.75 ± 0.21 bc	4.44 ± 0.08 a	4.40 ± 0.15 a	2.60 ± 0.08 c	2.65 ± 0.16 bc
K	58.8 ± 0.95 ab	44.0 ± 1.49 c	65.1 ± 2.4 a	53.4 ± 1.38 b	43.3 ± 1.05 c	40.13 ± 2.08 c
Ca	5.09 ± 0.33 b	5.04 ± 0.37 b	9.52 ± 0.10 a	9.78 ± 0.11 a	5.72 ± 0.20 b	5.77 ± 0.14 b
N	45.94 ± 1.6 bc	51.31 ± 0.90 a	43.51 ± 0.77 cd	49.38 ± 1.04 ab	40.43 ± 0.68 d	45.23 ± 0.32 bcd
S	4.33 ± 0.05 a	1.63 ± 0.02 e	3.89 ± 0.02 b	1.90 ± 0.02 d	3.83 ± 0.01 b	2.14 ± 0.008 c
N/S ratio	10.60 ± 0.27 d	31.40 ± 0.28 a	11.07 ± 0.23 d	25.16 ± 0.46 b	10.56 ± 0.16 d	21.01 ± 0.27 c
**Micronutrients**						
Mn	340.3 ± 1.3 c	255.0 ± 1.2 d	441.0 ± 1.4 a	415.5 ± 0.9 b	251.7 ± 0.5 d	238.3 ± 0.4 e
Fe	142.5 ± 1.9 a	132.8 ± 1.6 b	149.7 ± 1.1 a	147.5 ± 1.3 a	127.8 ± 1.9 b	128.0 ± 1.7 b
Cu	8.42 ± 0.16 c	8.57 ± 0.17 c	14. 89 ± 0.28 a	14.84 ± 0.19 a	12.0 ± 0.13 b	12.14 ± 0.25 b
Zn	78.49 ± 2.1 c	84.43 ± 2.5 bc	94.96 ± 1.0 b	110.80 ± 3.7 a	77.57 ± 1.3 c	74.87 ± 1.9 c

Data presented are the means ± SDs of four replicates. Different letters show statistically significant differences among all the treatments (*p* ≤ 0.05; Tukey’s test).

**Table 3:** Dereplicated metabolites from the three lettuce cultivars V1, V2 and V3 under +S and −S conditions. MW: Molecular weight, t_R_: Retention time, Pos.: Positive mode, Neg: Negative mode, Ref: Reference for dereplication.

Compound	MW	Class	Cultivar	*m*/*z*	tR (min)	Mode	Ref
Lactucin	276	Sesquiterpene lactone	V1	277.1	4.5	Pos.	[52]
Dihydrolactucopicrin	412	Sesquiterpene lactone	All	413.1	7.3	Pos.	[52]
Lactucopicrin	410	Sesquiterpene lactone	V1, V2, V3	411.1	7.3, 7.4	Pos.	[53]
Cyanidin 3-*O*-galactoside	448	Anthocyanin glycoside	V3	449.1	4.1	Pos.	[52]
Luteolin-7-glucuronide	462	Flavonoid-7-*O*-glucuronide	All	463.1	5.5	Pos.	[54]
Dicaffeoyltartaric acid (DCTA)	474	Phenylpropanoic acid ester	V1, V3, V2	472.9	5.3, 5.4	Neg.	[55]
Isoquercetin	464	Flavonoid	All	463.0	5.6	Neg.	[52]
Quercetin	302	Flavonoid	All	303.0	5.4	Pos.	[52]
Quercetin-3-*O*-glucose-6″-acetate	506	Flavonoid-3-*O*-glycoside	All	505.0	5.8	Neg.	[56]
Dicaffeoylquinic acid (DCQA)	516	Phenylpropanoic acid ester	V1, V3, V2	515.0	5.9, 5.8	Neg.	[55]
5-*O*-Caffeoylquinic acid (5-CQA)	354	Phenylpropanoic acid ester	V1, V2, V3	352.9	4.0, 3.9	Neg.	[57]
Caffeic acid hexose	342	Phenylpropanoid derivative	V1 V3	340.9 340.8	5.7, 5.8	Neg.	[58]
Quercetin 3-*O*-malonyl glucoside (QMG)	550	Flavonoid-3-*O*-glycoside	V1, V3, V2	551.1	5.6, 5.7	Pos.	[52]
Luteolin 3′,4′-di-*O*-β-d-glucopyranoside	610	Flavonoid glycoside	V1, V2, V3	611.0 611.2	5.0, 5.1	Pos.	[52]
Quercetin 3,4′-diglucoside	626	Flavonoid-3-*O*-glycoside	V1, V2, V3	627.1 627.1 627.2	4.1, 4.2, 4.2	Pos.	[52]
Kaempferol malonyl glucoside (KMG)	534	Flavonoid-3-*O*-glycoside	V3	535.1	4.8	Pos.	[59]

**Table 4:** Fold change (−S/+S) and *p*-values of these 14 identified metabolites of butterhead green lettuce (V1) and multi-leaf green lettuce (V2).

Metabolite	FC	Log2 (FC)	Raw *p* Value	−log10(*p*)
**Butterhead green lettuce (Pazmanea RZ, V1)**				
Luteolin-7-*O*-glucuronide	5.8241	2.542	4.31 × 10^−13^	12.365
Quercetin 3,4′-diglucoside	30.015	4.9076	3.98 × 10^−10^	9.3999
Quercetin-3-*O*-glucose-6′′-acetate	12.783	3.6761	1.07 × 10^−9^	8.9706
Isoquercetin	7.0765	2.823	1.08 × 10^−9^	8.9659
Quercetin	11.674	3.5453	9.76 × 10^−9^	8.0105
Caffeic acid hexose	0.056472	−4.1463	7.68 × 10^−7^	6.1145
5-*O*-Caffeoylquinic acid (5-CQA)	5.5566	2.4742	1.40 × 10^−6^	5.8541
Quercetin 3-*O*-malonylglucoside (QMG)	6.5456	2.7105	3.21 × 10^−5^	4.493
Luteolin 3′,4′-di-*O*-β-d-glucopyranoside	3.3714	1.7533	0.00015003	3.8238
Dicaffeoyltartaric acid (DCTA)	1.7986	0.84687	0.00072512	3.1396
Dicaffeoylquinic acid (DCQA)	1.8121	0.85762	0.001202	2.9201
Dihydrolactucopicrin	2.9272	1.5495	0.0033116	2.48
Lactucin	2.7155	1.4412	0.028758	1.5412
Lactucopicrin	1.1981	0.26077	0.1786	0.74811
**Multi-leaf green lettuce (Hawking RZ, V2)**				
Luteolin-7-*O*-glucuronide	1.1586	0.21234	0.15066	0.82201
Quercetin 3,4′-diglucoside	2.1049	1.0738	1.04 × 10^−5^	4.9828
Quercetin-3-*O*-glucose-6″-acetate	1.8942	0.92157	0.0020218	2.6943
Isoquercetin	1.6636	0.73435	3.68 × 10^−6^	5.4341
Quercetin	1.7305	0.79122	7.44 × 10^−5^	4.1284
5-*O*-Caffeoylquinic acid (5-CQA)	2.2622	1.1778	2.89 × 10^−5^	4.5399
Quercetin 3-*O*-malonylglucoside (QMG)	1.4393	0.52532	0.038325	1.4165
Luteolin 3′,4′-di-*O*-β-d-glucopyranoside	1.0201	0.028749	0.73936	0.13114
Dicaffeoyltartaric acid (DCTA)	1.1952	0.25728	0.13878	0.85767
Dicaffeoylquinic acid (DCQA)	1.5972	0.67556	0.00091261	3.0397
Dihydrolactucopicrin	1.9898	0.99263	0.0047081	2.3272
Lactucopicrin	2.4406	1.2872	0.0020288	2.6928

**Table 5:** Antibacterial activity (%) against *S. aureus* at test concentrations of 200 µg/ml and DPPH radical scavenging activity of three lettuce cultivars V1–V3 grown under both +S and −S conditions.

Treatments	Antibacterial Activity %	DPPH IC_50_ (µg/mL)
V1/+S	39.06 ± 7.8a	>200a
V1/−S	n.d	>200a
V2/+S	39.06 ± 7.8a	>200a
V2/−S	n.d	>200a
V3/+S	44.34 ± 5.41a	60.53 ± 0.06b
V3/−S	43.55 ± 7.34a	62.83 ± 1.29b
Chloramphenicol	98 at 3 μg/mL	
Ascorbic acid		4.1 ± 0.0

Data presented are the means ± SDs of four replicates. Different letters show statistically significant differences among all the treatments (*p* ≤ 0.05; Tukey’s test).

## Data Availability

Not applicable.

## Supplementary Materials

The following are available online at https://www.mdpi.com/article/10.3390/pharmaceutics13050713/s1, Figure S1: Detailed quantitative analysis of compounds detected in multi-leaf green lettuce (V2) grown under +S and −S conditions, Figure S2: Detailed quantitative analysis of compounds detected in multi-leaf red lettuce (V3) grown under +S and −S conditions.
