# Supplementary material for: Comparative Metabolite Profile, Biological Activity and Overall Quality of Three Lettuce (Lactuca sativa L., Asteraceae) Cultivars in Response to Sulfur Nutrition
Source: Pharmaceutics. 2021 May 13;13(5):713. doi: 10.3390/pharmaceutics13050713 (PMC8153342; doi:10.3390/pharmaceutics13050713)
Supplement: Supplementary file 1 [file pharmaceutics-13-00713-s001.zip › pharmaceutics-1198751-supplementary.pdf]

pharmaceutics

MDPI

Supplementary Materials: Comparative Metabolite Profile, Biological Activity and Overall Quality of Three Lettuce (*Lactuca sativa* L., Asteraceae) Cultivars in Response to Sulfur Nutrition

Muna Ali Abdalla, Fengjie Li, Arlette Wenzel-Storjohann, Saad Sulieman, Deniz Tasdemir and Karl H. Mühling

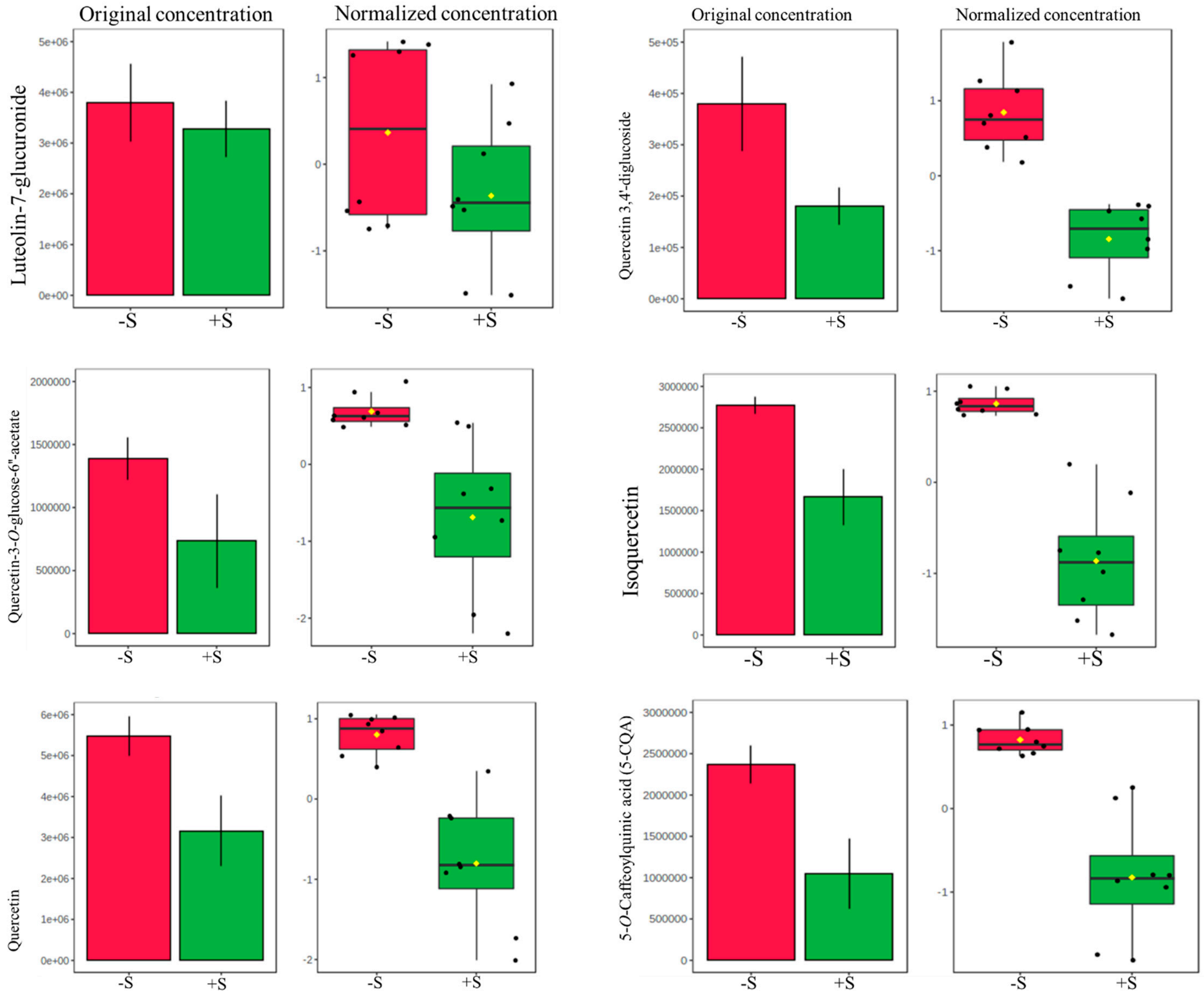

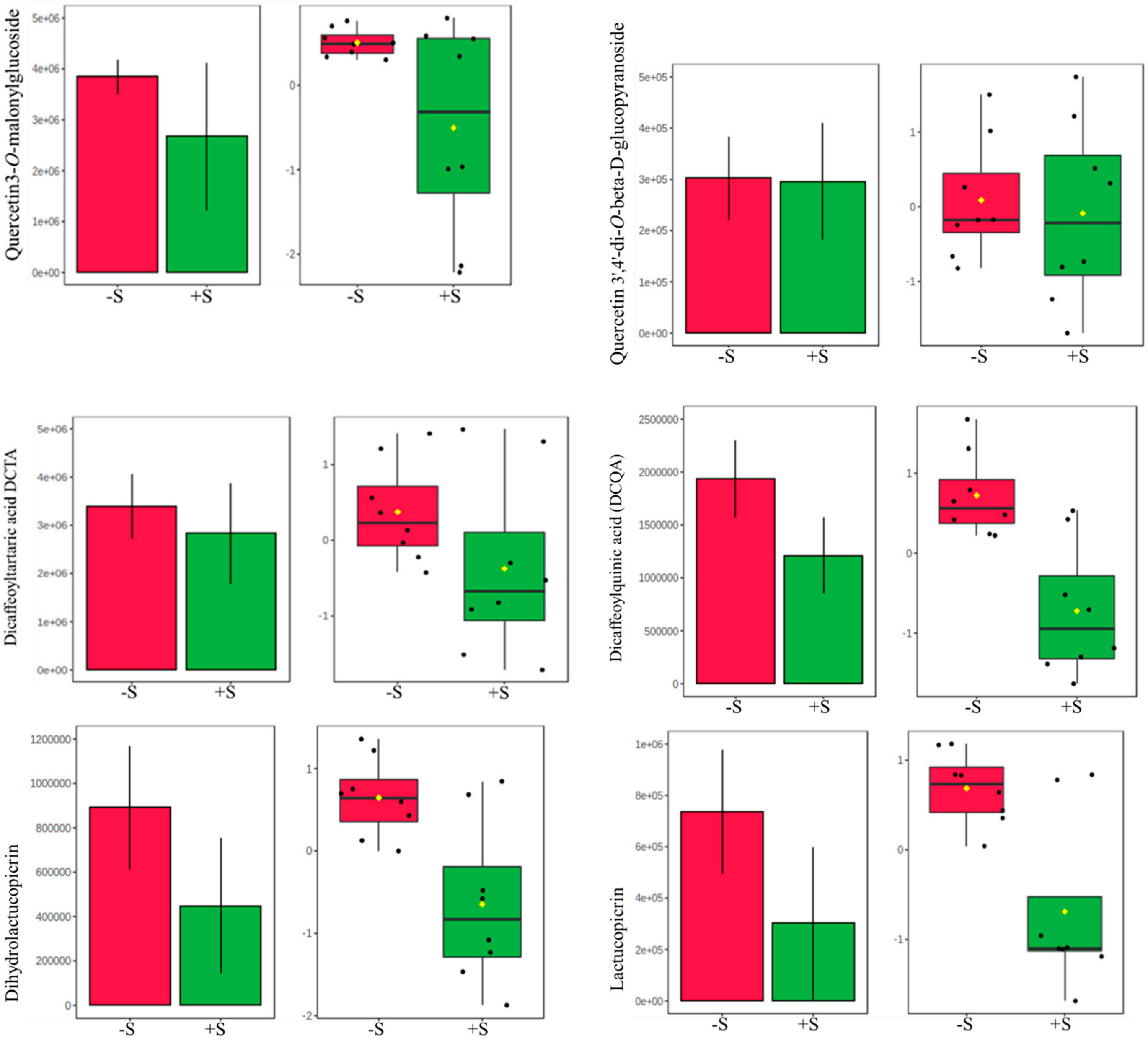

Figure S1. Detailed quantitative analysis of compounds detected in multi-leaf green lettuce (V2) grown under +S and −S conditions.

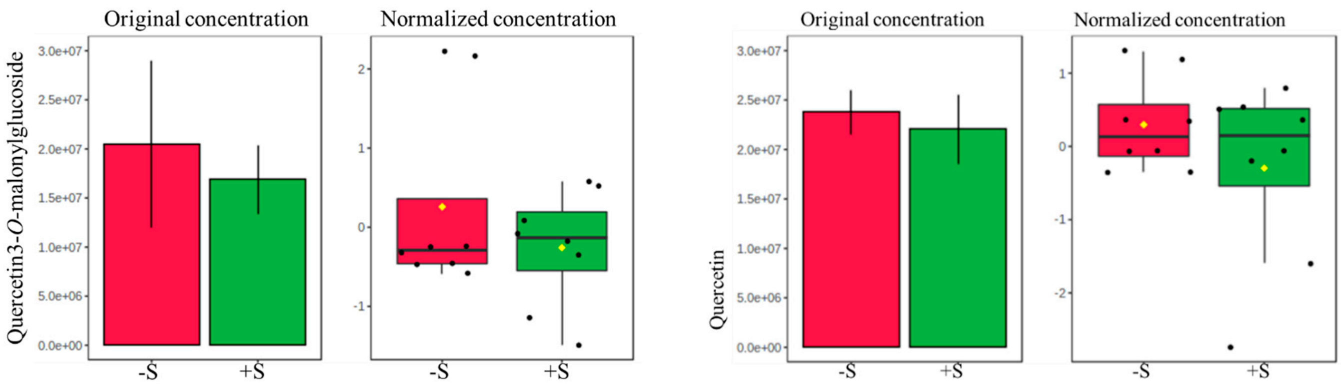

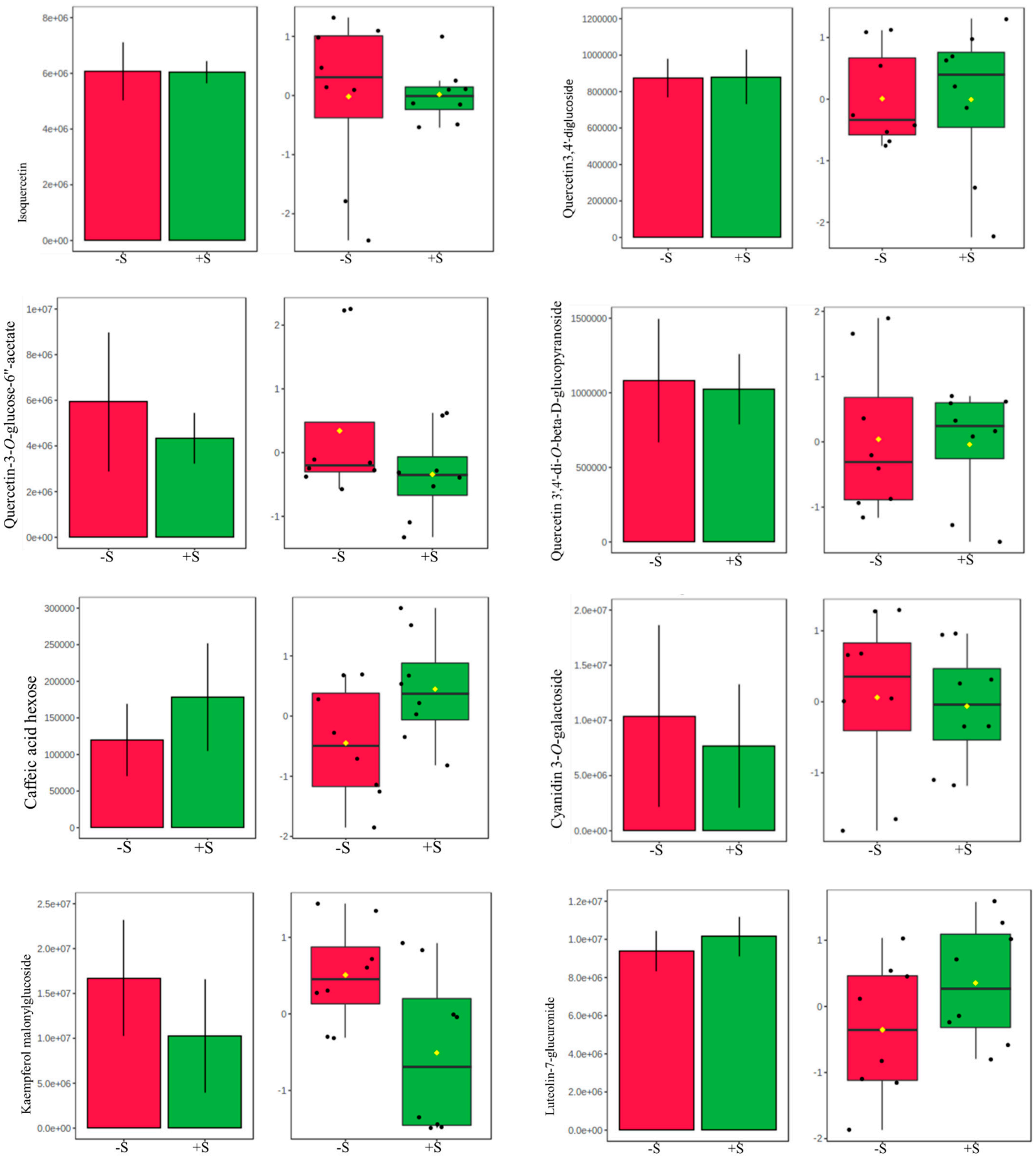
Isoquercetin
8e+06
6e+06
4e+06
2e+06
0e+00
-S
+S
1
0
-1
-2
-S
+S
Quercetin3,4'-diglucoside
1200000
1000000
800000
600000
400000
200000
0
-S
+S
1
0
-1
-2
-S
+S
Quercetin-3-O-glucose-6''-acetate
1e+07
8e+06
6e+06
4e+06
2e+06
0e+00
-S
+S
2
1
0
-1
-S
+S
Quercetin 3',4'-di-O-beta-D-glucopyranoside
1500000
1000000
500000
0
-S
+S
2
1
0
-1
-S
+S
Caffeic acid hexose
300000
250000
200000
150000
100000
50000
0
-S
+S
1
0
-1
-2
-S
+S
Cyanidin 3-O-galactoside
2.0e+07
1.5e+07
1.0e+07
5.0e+06
0.0e+00
-S
+S
1
0
-1
-S
+S
Kaempferol malonylglucoside
2.5e+07
2.0e+07
1.5e+07
1.0e+07
5.0e+06
0.0e+00
-S
+S
1
0
-1
-S
+S
Luteolin-7-glucuronide
1.2e+07
1.0e+07
8.0e+06
6.0e+06
4.0e+06
2.0e+06
0.0e+00
-S
+S
1
0
-1
-2
-S
+S

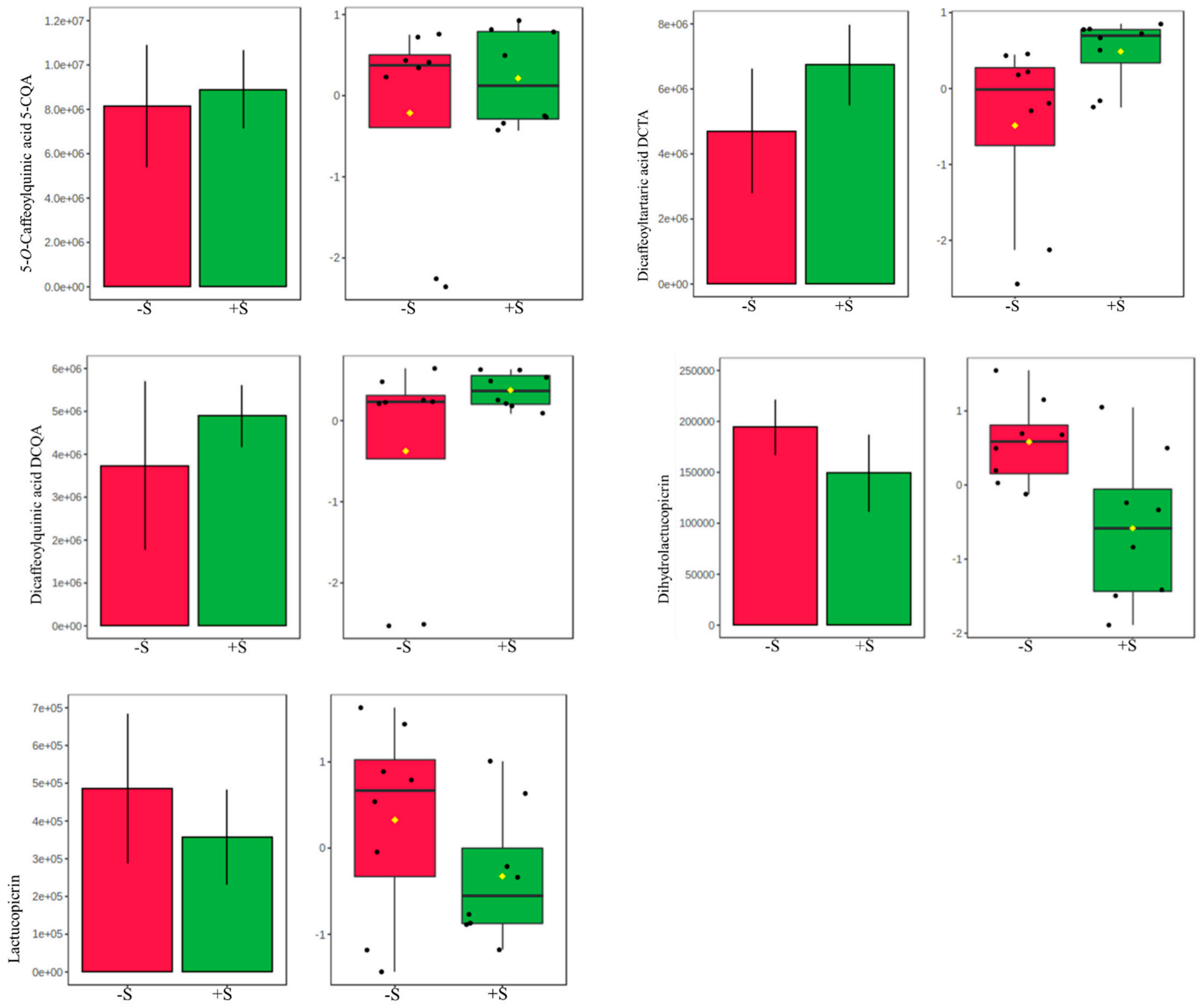

Figure S2. Detailed quantitative analysis of compounds detected in multi-leaf red lettuce (V3) grown under +S and −S conditions.
